# The good, the bad, and the mixed: Experiences during COVID-19 among an online sample of adults

**DOI:** 10.1371/journal.pone.0269382

**Published:** 2022-06-24

**Authors:** Devin J. Mills, Julia Petrovic, Jessica Mettler, Chloe A. Hamza, Nancy L. Heath

**Affiliations:** 1 Department of Community, Family, and Addiction Sciences, Texas Tech University, Lubbock, Texas, United States of America; 2 Department of Educational and Counselling Psychology, McGill University, Montreal, Canada; 3 Department of Applied Psychology and Human Development, University of Toronto, Toronto, Canada; Univerzitet u Beogradu Filozofski Fakultet, SERBIA

## Abstract

Studies have outlined the negative consequences of the COVID-19 pandemic to psychological health. However, the potential within-individual diversity of experiences during COVID-19, and how such experiences relate to indices of psychological distress and COVID-19-specific stressors, remains to be explored. A large online sample of American MTurk Workers (*N* = 3,731; *M*_age_ = 39.54 years, *SD* = 13.12; 51.70% female) completed short assessments of psychological distress, COVID-19-specific stressors (e.g., wage loss, death), and seven items assessing negative and positive COVID-19 experiences. Latent profile analyses were used to identify underlying profiles of COVID-19 experiences. A four-profile solution was retained representing profiles that were: (1) predominantly positive (*n* = 839; 22.49%), (2) predominantly negative (*n* = 849; 22.76%), (3) moderately mixed (*n* = 1,748; 46.85%), and (4) high mixed (*n* = 295; 7.91%). The predominantly positive profile was associated with lower psychological distress, whereas both the predominantly negative and high mixed profiles were associated with higher psychological distress. Interestingly, specific COVID-19 stressful events were associated with the high mixed profile. The present study challenges the narrative that the impacts of COVID-19 have been unilaterally negative. Future directions for research are proposed.

## Introduction

The COVID-19 pandemic, along with the unprecedented series of lockdowns that followed its onset, caused drastic disruptions to day-to-day life globally. A substantial proportion of students and employees experienced a sudden shift to remote work and lockdown measures abruptly halted social activities, leading to increased social isolation [1, 2]. Accordingly, to date, there are multiple studies which have focused on the negative impacts of the COVID-19 pandemic onset, with several highlighting aversive psychological impacts [3–5]. However, it has been suggested that an overemphasis on the negative consequences of COVID-19 may bring about expectancy effects [6] and the capacity for resilience in response to COVID-19 cannot be discounted in our investigations of the impact of the pandemic [7, 8]. Moreover, a balanced exploration into both negative and positive experiences during early months of the pandemic, their potential for co-occurrence, and whether they differ as a function of COVID-19-specific COVID-19 stressful events remains to be examined. Thus, the present study took a person-centered approach to examine the potential variability in COVID-19 experiences among a large and diverse sample of adults, and to investigate how differences in specific COVID-19-related stressors (i.e., loss of wages, COVID-19 diagnosis of self or loved one, death of loved one due to COVID-19) were associated with COVID-19 experiences.

### Negative experiences during COVID-19

During the early months of COVID-19, numerous studies reported on the negative impacts and stressful events associated with the first series of global lockdowns. Here, we distinguish between psychological experiences during COVID-19 (e.g., feeling stressed, sad, or lonely) and COVID-19 stressful events (e.g., job loss, death). Heightened levels of mental health difficulties were commonly reported (e.g., distress, anxiety, depression) [4, 5, 9, 10]. However, emerging longitudinal research suggests these effects may be small, and there is likely considerable variability in the psychological experiences of COVID-19 [6]. Nevertheless, psychological harms, concern towards the health of vulnerable loved ones, as well as loss of leisure and health activities, were commonly reported during the early months of COVID-19 [2, 11].

Other studies have focused on the mental health disparities associated with COVID-19. For instance, two studies of individuals with eating disorders found that disordered eating behaviours were exacerbated in the first few weeks of the pandemic [12, 13]; these were among a number of studies to report that COVID-19 exacerbated the struggles of groups experiencing various difficulties prior to the onset of the pandemic. In another early pandemic study, Iob and colleagues [14] found that COVID-19 instigated an amplification of pre-existing inequalities among disadvantaged groups including ethnic minority groups, those experiencing socioeconomic disadvantages, and the unemployed. Furthermore, in a qualitative study of distress and coping in India during the first COVID-19 lockdown, disadvantaged groups with limited access to mobile phones, health messaging, or health care experienced extreme distress and despair, greater health needs, loss of income, and further social exclusion as a result of the pandemic [10]. Taken together, these findings suggest that COVID-19 may have worsened existing psychosocial and financial inequalities [5, 15].

Social isolation has been commonly reported as a primary cause of increased psychological distress among some individuals during COVID-19 [11]. One longitudinal study of university students revealed that individuals without pre-existing mental health concerns were more likely than individuals with pre-existing mental health concerns to experience declining mental health during the early months of the pandemic, which corresponded with increased social isolation among these students (whereas there was no change for students with pre-existing mental health concerns) [16]. Other contextual factors that have been found to be contributors to negative COVID-19 experiences among general population samples during the early months of the pandemic include economic fallout (e.g., wage loss), grief from having lost a loved one to COVID-19, trauma associated with surviving COVID-19, the inability to see relatives (especially older relatives), having to manage the impracticalities of working or schooling from home, the disruption of social and recreational activities, and frustration with the media or government [2, 17]. In short, much of the literature has focused on the negative consequences associated with COVID-19 events without accounting for the possibility of positive experiences emanating from COVID-19 events.

### Positive COVID-19 experiences

Despite the numerous negative psychological experiences of the onset of COVID-19 on populations worldwide, preliminary findings have shed light on the possibility that the experiences of COVID-19 during its early months have not been unanimously negative [6, 18]. For example, a study by Pinkham and colleagues [19] of individuals with severe mental illness found that their affective experiences and psychotic symptoms remained stable throughout the early months of the pandemic, and that they actually experienced an increase in well-being during this timeframe. Additionally, a longitudinal study with over 50,000 UK adults found that across early pandemic months, individuals with pre-existing mental health conditions experienced significantly greater decreases in anxiety than individuals without pre-existing mental health conditions [20]. These findings suggest that in certain instances, the onset of COVID-19 brought about positive, rather than negative, psychological impact for some individuals.

A limited number of studies have explored positive experiences during the onset of the pandemic among more general populations. Indeed, research suggests that the shared experience of the pandemic may have strengthened social connectedness, since people reported feeling as though “we are all in this together” [6, 21, 22]. Even among disadvantaged groups who report a disproportionate degree of negative COVID-19 experiences, themes of resilience and healthy coping have emerged in reports of their experiences, which have included reports of social connectedness as well as finding sense and meaning in the pandemic [10]. Typically, these studies have been limited in the breadth of positive experiences examined.

To our knowledge, only four studies have specifically focused on the investigation of a broad range of positive experiences during early COVID-19 months among general community samples. The first was a cross-sectional online study by Stallard and colleagues [23] which investigated positive COVID-19 experiences reported by parents and caregivers (88.6% of the sample were mothers) in Portugal and the UK during the first lockdown. It was found that as many as 88.6% of participants identified positives arising from COVID-19 within an open-ended question. A second cross-sectional study by Williams and colleagues [18] conducted in Scotland during the first lockdown explored positive changes experienced during COVID-19, and the underlying sociodemographic predictors of such changes. Again, the majority of participants reported positive changes including having more quality time with their partner (53.3%), to be in nature (65.2%), do enjoyable activities (67.4%), and exercise (53.9%). Participants also reported being more appreciative of things usually taken for granted (82.6%). A third study by Schmiedeberg and Thönnissen [24] of German adults explored the extent to which individuals held positive and/or negative perceptions regarding COVID-19. Using two items, one for positive perception and one for negative perception, the authors found 61% agreed with positive perceptions towards COVID-19 (i.e., being able to see the positive sides of the pandemic), whereas only 26% agreed with negative perceptions (i.e., feeling strongly affected by the pandemic). Finally, Hampshire and colleagues [2] investigated positive and negative COVID-19 experiences during May 2020 among a large sample (*N* > 100,000) of participants aged 16 to 85 and older, as well as sociodemographic and neurological/psychiatric predictors of such experiences. Participants reported strong endorsement of a number of positive COVID-19 experiences (i.e., improved natural environment, enjoying the simpler things in life, spending less money, and a greater sense of community) as well as of a number of negative COVID-19 experiences (i.e., loss of leisure/health activities, and concern for health of loved ones, which was higher than concern about one’s own health). Furthermore, sociodemographic characteristics, work, environment, and social circumstances revealed robust associations with the nature and extent of self-reported positive and negative COVID-19 experiences.

Taken together, the studies outlined above highlight the importance of measuring multiple dimensions of both negative and positive COVID-19 experiences when quantifying the breadth of impacts of the onset of COVID-19. Yet, much of the available research presents a view of the effect of COVID-19 as all negative, and research that has assessed the positive outcomes of COVID-19 is limited. Hampshire and colleagues [2] assessed a comprehensive overview of both positive and negative experiences revealing a sufficient number of participants endorsed both positive and negative. This is due to past research primarily using variable-centered approaches, which help to reveal general associations among variables but fail to account for individual variability across a set of variables. As a result, researchers have yet to examine whether there are subgroups of individuals, based on the scores on both positive and negative indicators (e.g., someone who is strongly positive, but also somewhat negative). A person-centered analysis takes into account this heterogeneity.

### The present study

The present study sought to explore the range of experiences during the COVID-19 pandemic among an online community sample of adults, as well as whether COVID-19-specific stressful events were related to such experiences. The first objective was thus to investigate the factor structure of a researcher-designed measure of negative and positive COVID-19 experiences and its convergent and divergent validity with psychological distress markers (i.e., stress, anxiety, depression). Given the paucity of work exploring the potential positives of COVID-19, developing this scale represents an important contribution to the emerging literature on the effects of COVID-19 on individuals. Based on previous literature, we hypothesized that two distinct factors would emerge: one for negative COVID-19 experiences and one for positive COVID-19 experiences. Furthermore, we anticipated that negative experiences during COVID-19 would be associated with greater stress, anxiety, and depression. Conversely, positive experiences related to COVID-19 were expected to be associated with less stress, anxiety, and depression. Given that many authors have suggested that impacts of COVID-19 will continue long after the pandemic has ended, [25], this scale can continue to be used to explore both positive and negative experiences resulting from the pandemic in the years to come. However, it is also expected that the measure may be easily adapted to assess positive and negative experiences to other global, national, or local crises (e.g., political unrest, natural disasters).

The second objective of this study was to use a person-centered approach to identify underlying profiles of COVID-19 experiences, taking into account the diversity of negative and positive experiences. Contrary to a variable-centered approach, the person-centered approach is expected to yield more information regarding the underlying relationship of both negative and positive COVID-19 experiences within individuals. Finally, the third objective sought to better understand the emergent profiles by comparing them in terms of differences in demographic information, psychological distress, and specific stressful events during COVID-19 (i.e., loss of wages, diagnosis of self or other, knowing someone who died from COVID-19). Given the inherently exploratory nature of this approach, no specific hypotheses were made for these last two objectives.

## Methods

### COVID-19 context

The first COVID-19 case in the United States was confirmed by the Centers for Disease Control and Prevention on January 21, 2020, and a Public Health Emergency was declared within two weeks on February 3, 2020. On March 12, 2020, financial markets were down nearly 10% in the United States with rising concerns of business closings. By late March, many states enacted stay-at-home orders, and employees began to be furloughed. Unemployment within the US jumped from less than 5% in February to nearly 15% by April. Although cases continued to rise in the United States, many state-mandated stay-at-home orders expired by the end of May. As of June 2020, the financial challenges were amplified by the rising health concerns as the confirmed COVID-19 case count in the United States exceeded 2 million with more than 100,000 deaths related to complications with COVID-19. This represents the context in which the present data was collected. Data for the present study are available online here: https://tinyurl.com/m54cfraf.

### Participants and procedure

The Texas Tech University Review Board (IRB2019-920) approved the present study. Participants were provided information about the study prior to completing the online survey anonymously. As part of a larger study on the mental health and risky behaviors of workers on Amazon’s Mechanical Turk (MTURK), a short, five-minute survey was posted on MTURK to screen participants for future research. MTURK has been used extensively in social sciences with research demonstrating benefits including cost effectiveness, speed, and data quality [26, 27]. The survey was created in Qualtrics and posted on MTURK via the TurkPrime.com platform [28] which, in addition to being user friendly, offers additional services including blocking known problem workers and bots. Participants provided their informed consent following a review of the intended research goals and continued to the online survey.

The survey was open for a seven-day period from June 8, 2020 to June 14, 2020 and was only open to participants with ≥ 90% approval in at least 100 previous assignments on MTURK. Participants were paid $0.50. In total, 4,771 participants initiated the survey on MTURK. Participants were excluded for missing one or more attention item (*n* = 640), engaging in “straight lining” (*n* = 226), or submitting missing data (*n* = 174). The final sample included 3,731 participants (*M*_age_ = 39.54 years, *SD* = 13.12; 51.70% female) (see Table 1 for sample demographics).

**Table 1:** Sample demographics and descriptives statistics for mental health indices.

**Sample Demographics**	***n***	***%***
**Sex**		
Male	1830	49.049%
Female	1901	50.951%
**Race / Ethnicity**		
Caucasian or White	2723	72.983%
Hispanic or Latino	171	4.583%
African American or Black	317	8.496%
Asian American or Asian	337	9.032%
Multiracial	183	4.905%
**Highest Education Level**		
High school diploma or equivalent including GED	294	7.880%
Some college but no degree	655	17.556%
Associate degree in college (2-year)	345	9.247%
Bachelor’s degree in college (4-year)	1675	44.894%
Master’s degree	630	16.886%
Doctoral degree	70	1.876%
Professional degree (JD, MD)	62	1.662%
**Employment prior to COVID-19**		
Working	3018	80.890%
Laid off or Looking for work	266	7.129%
Retired, Disabled or Otherwise not working	447	11.981%
**Annual Income**		
Less than $29,999	940	25.194%
$30,000 to $49,999	858	22.997%
$50,000 to $99,999	1442	38.649%
$100,000 or more	491	13.160%
**Sample Descriptives**	***M***	***SD***
Stress (PSS-4; Past Month)	6.47	3.47
Anxiety (GAD-2; Past Two Weeks)	1.83	1.81
Depression (PGQ-2; Past Two Weeks)	1.67	1.72

### Measures

#### Negative and positive experiences during COVID-19

Seven items were created for the purposes of the present study based on previous exploratory, qualitative interviews with adults regarding their experiences during the early months of COVID-19. Items followed the prompt, “*Relative to months before COVID-19*…” and were rated on a 6-point scale ranging from *Strongly Disagree* (1) to *Strongly Agree* (6). The brevity of the scale was intentional as it reduces participant burden and would be scalable for future large-scale studies including continued COVID-19 research. Further, a briefer measure would make it more useful in clinical contexts. Table 2 presents these items and the item descriptives.

**Table 2:** Items descriptives and loadings from the exploratory (EFA) and confirmatory (CFA) factor analyses on the COVID-19 experience items.

	Item Descriptives		EFA		CFA	
			Loadings		Loadings	
	*M*	*SD*	F1	F2	Negative	Positive
**COVID-19 Experience Items** (*Relative to months before COVID-19…*)						
I have been more stressed.	3.91	1.39	0.58	-0.22	0.82	
I have felt closer to others.	3.33	1.36	0.07	0.55		0.53
I have been less anxious or worried.	2.83	1.38	0.05	0.66		0.64
I have more time to do things I enjoy.	3.76	1.33	0.01	0.52		0.52
I have been lonelier.	3.38	1.52	0.72	0.02	0.62	
I have felt sad or down more.	3.45	1.50	0.98	0.03	0.82	
I have felt happier.	3.14	1.33	-0.06	0.83		0.89

#### Demographics

Basic demographics were collected including sex, age, race, level of education, and income. Due to the timing of data collection within the United States, it was possible that some individuals were experiencing complicated circumstances regarding their employment status; as a result, participants reported their employment prior to COVID-19.

#### Specific COVID-19 events

Three Yes/No questions were asked regarding specific COVID-19 events: (1) *Have you lost wages because of the COVID-19 pandemic*?; (2) *Have you or someone you know been diagnosed with the coronavirus (COVID-19)*?; and (3) *Has someone you know died due to complications with the coronavirus (COVID-19)*?

#### Stress, depression, and anxiety

Short forms of psychological distress measures were selected due to space restrictions. The Perceived Stress Scale (PSS) [29] was used to assess subjective experiences of stress during the past month. Items were rated on 5-point scale ranging from *Never* (0) to *Very Often* (4). The internal consistency of the PSS-4 was 0.81 in the present study. Two-item versions of the Patient Health Questionnaire (PHQ2) [30] and the General Anxiety Disorder scale (GAD2) [31] were used to assess depressive and anxiety symptoms, respectively. For both scales, participants rated the occurrence of symptoms over the past two weeks on a scale ranging from *Not at all* (0) to *Nearly every day* (3), with higher scores indicating greater levels of depression and anxiety. The correlation between the two PHQ2 items was 0.78, and between the two GAD2 items was 0.75. Sample descriptives are present in Table 1.

### Data analysis

Data were cleaned and descriptive statistics computed in SPSS version 26 [32]. Mplus version 8 [33] was used evaluate the factor structure of the seven created items assessing negative and positive experiences related to COVID-19. After randomly splitting the sample in half, data from the first group (*n* = 1,888; *M*_age_ = 39.54 years, *SD* = 13.06; 51.70% female) were used within an exploratory factor analysis (EFA) with Promax rotation. Data from the second group (*n* = 1,843; *M*_age_ = 39.40, *SD* = 13.17; 50.19% female) were used in a confirmatory factor analysis (CFA) with robust maximum likelihood to further confirm the observed factor structure observed within the EFA. Several goodness-of-fit indices were used to assess the fit of the data to the resulting factor structure including the root mean square error of approximation (RMSEA; ≤ .05), comparative fit index (CFI; ≥ .90), Tucker-Lewis Index (TLI; ≥ .90), and standardized root mean squared residual (SRMR; ≤ .06) [34].

Subsequently, a series of latent profiles (LPAs) were conducted using the seven individual items with means and variances freely estimated [35]. All models were estimated with 5000 random start values, 1000 iterations, and the 200 best solutions were retained. Starting with a model with one profile, model with an increasing number of profiles were considered. Values for the Akaike Information Criterion (AIC), the Bayesian Information Criterion (BIC), and the Sample-Size-Adjusted BIC (SSABIC) graphed following each LPA in order to identify the point at which decreases in these information criteria begin to plateau (i.e., the “elbow”) [35, 36].

The adjusted Lo-Mendell-Rubin likelihood ratio test (aLRT) was conducted for models with 2 or more profiles, which tests whether the model with *k* profiles is preferred to one with to *k–* 1 profiles. A significant result suggests preference for the model with *k* profiles [37]. Although not used in the selection process [38, 39], entropy is commonly reported as it provides an assessment of the precision classification. Entropy values range from 0 (high uncertainty) to 1 (low uncertainty).

## Results

### Exploratory and confirmatory factor analysis

The resulting eigenvalues from the EFA with Promax rotation for the first two factors were 2.94 (Factor 1) and 1.49 (Factor 2) and accounted for 42.00% and 21.29% of the variance, respectively. The eigenvalues for the remaining factors were less than 0.80 and not considered. Loadings from the EFA are presented in Table 2. Factor 1 represented negative COVID-19 experiences (e.g., I have been more stressed), whereas Factor 2 represented positive COVID-19 experiences (e.g., I have felt happier). The two factors were modestly negatively correlated (*r* = -0.38). Results from CFA revealed a slightly below adequate fit of the data to this 2-factor model (*χ*^2^ (13) = 202.45, *p* < .001; RMSEA = 0.09 90%CI[0.08, 0.10]; CFI = 0.94; TLI = 0.91; SRMR = 0.04). After reviewing the modification indices, error terms were allowed to correlate between item 2 and item 5 and between item 5 and item 6, which improved fit to an acceptable level (*χ*^2^ (11) = 115.04, *p* < .001; RMSEA = 0.07 90%CI[0.06, 0.08]; CFI = 0.97; TLI = 0.94; SRMR = 0.04). Loadings from the CFA are presented in Table 2. The correlation between negative and positive COVID-19 experiences was -0.51, *p* < 0.001.

Data from the entire sample were used within a measurement model in which negative and positive COVID-19 experiences (as modelled in the CFA) were covaried with composite scores on the PSS4, PHQ2, and GAD2, thus providing estimates of the bivariate correlations between the two COVID-19 experiences and the three indices of psychological distress through the TECH4 function of Mplus. Data fit the model adequately (*χ*^2^(26) = 535.30, *p* < .001; RMSEA = 0.07 90% CI [0.06, 0.08]; CFI = 0.96; TLI = 0.93; SRMR = 0.03). Results largely supported expectations in that negative COVID-19 experiences were positively associated with scores on the PSS4 (*r* = 0.62, *p* < .001), PHQ2 (*r* = 0.62, *p* < .001), and GAD2 (*r* = 0.62, *p* < .001), whereas positive COVID-19 experiences would be negatively associated with scores on the PSS4 (*r* = -0.30, *p* < .001), PHQ2 (*r* = -0.17, *p* < .001), and GAD2 (*r* = -0.15, *p* < .001).

### Latent profile analysis

**Fig 1:**
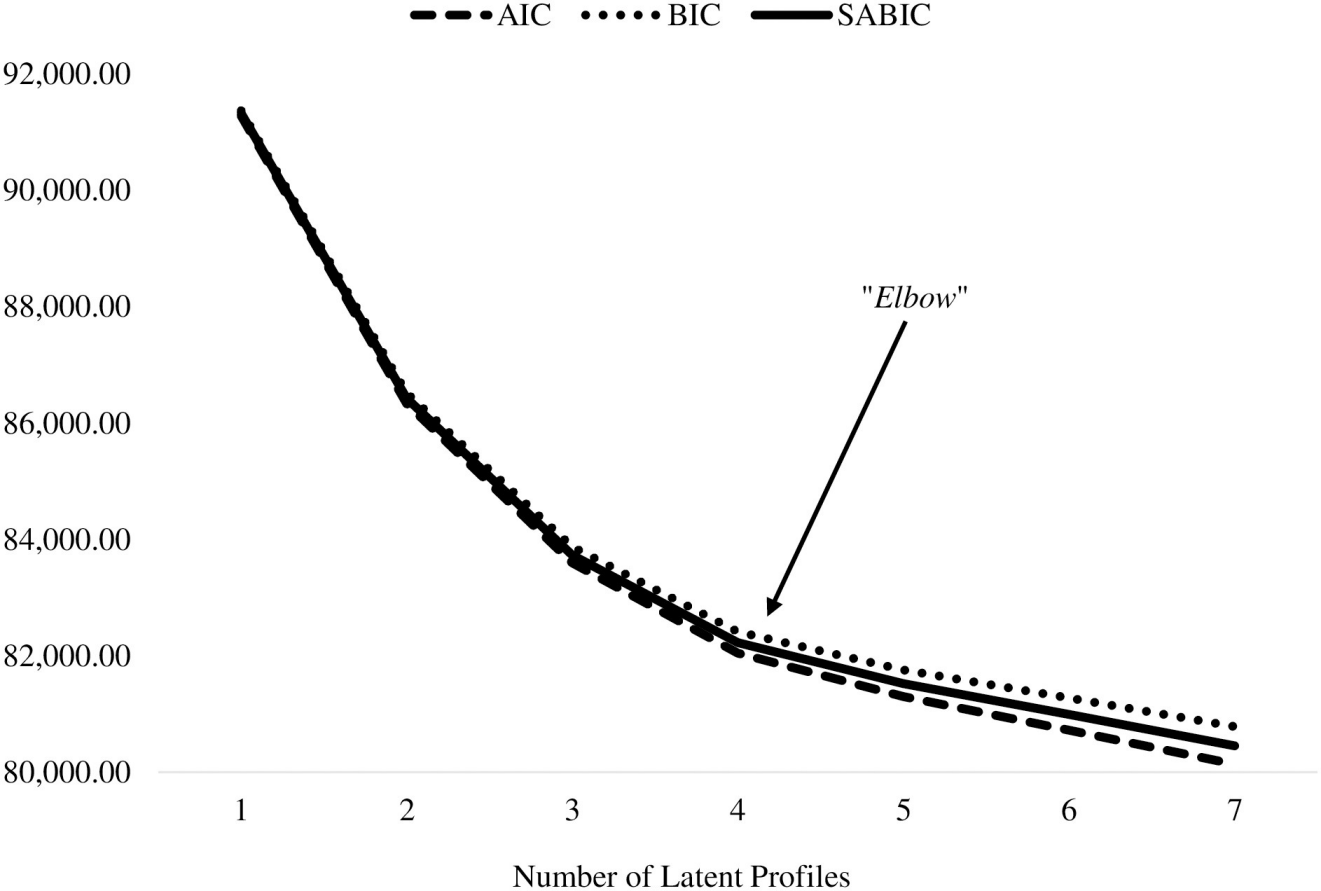
Values of the Akaike Information Criterion (AIC), the Bayesian Information Criterion (BIC), and the Sample-Size-Adjusted BIC (SSABIC) across the seven competing latent profile analyses.

Table 3 presents the model selection indices for each of the seven profiles. Values for the AIC, BIC, and SABIC were graphed in order to identify the point at which the decline in values plateaued (i.e., the “elbow”; see Fig 1). This was observed following the rise from four to five profiles suggesting a preference for the four-profile model. This was supported by the significant aLRT. Nonetheless, significant aLRTs also support both five- or six-profile models. As such, the five- and six-profile models were considered but found to result in largely uninterpretable profiles. Therefore, the four-profile model was selected.

**Table 3:** Model selection indices for latent profiles analyses.

	*AIC*	*BIC*	*SABIC*	*Entropy*	*aLRT*
1 Profile	91,277.69	91,364.83	91,320.35	n/a	n/a
2 Profiles	86,335.00	86,515.51	86,423.37	0.83	p < .001
3 Profiles	83,597.16	83,871.04	83,731.23	0.85	p < .001
**4 Profiles** ^a^	**82,044.63**	**82,411.88**	**82,224.40**	**0.86**	**p < .001**
5 Profiles	81,296.63	81,757.24	81,522.10	0.85	0.040
6 Profiles	80,720.35	81,274.32	80,991.52	0.84	0.001
7 Profiles	80,136.11	80,783.46	80,452.99	0.81	0.444

^a^ Selected model.

**Fig 2:**
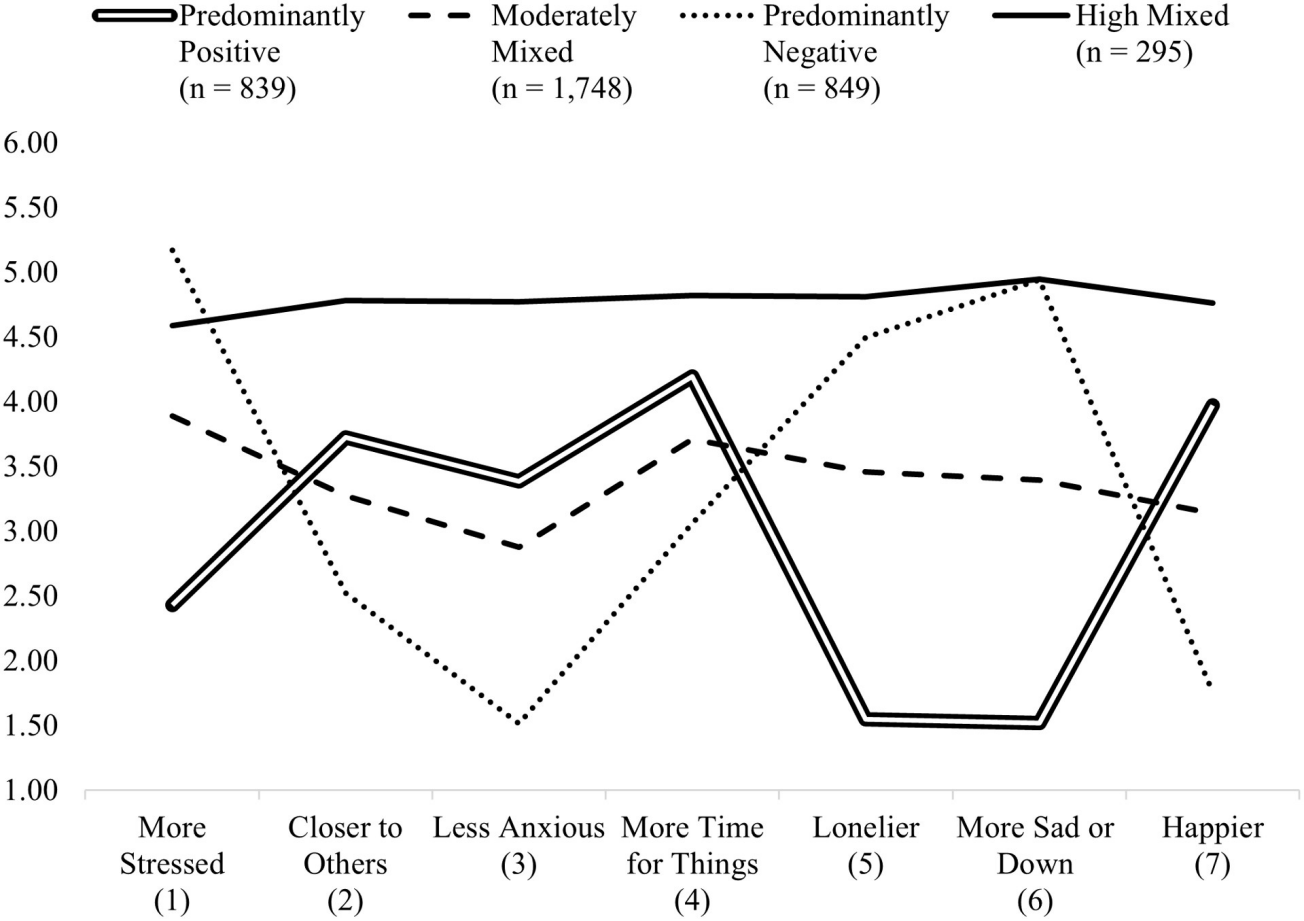
Means for the seven items across each of the four profiles.

Fig 2 presents the means of the seven items across each of the four profiles. Profile 1 (*n* = 839; 22.49%) is the “Predominantly Positive” profile representing those who more strongly endorsed positive versus negative experiences related to COVID-19. Profile 2 (*n* = 1,748; 46.85%) is the “Moderately Mixed” profile representing those who moderately endorsed both positive and negative experiences related to COVID-19. Profile 3 (*n* = 849; 22.76%) is the “Predominantly Negative” profile representing those who more strongly endorsed negative experiences related to COVID-19. Profile 4 (*n* = 295; 7.91%) is the “High Mixed” profile representing those who strongly endorsed both positive and negative experiences related to COVID-19.

Table 4 presents demographics across the four profiles. Significant differences, albeit small based on Cramer’s V (see note in tables), were found across sex, race/ethnicity, employment prior to COVID-19, and annual income (*p*’s < 0.001). Bonferroni-adjusted post-hoc comparisons were conducted to further explore the differences in the proportions. Results revealed that males were slightly over-represented in the Moderate Mixed and High Mixed profiles, whereas females were over-represented in the predominantly negative profile. Differences in race were largely attributed to an over-representation of Hispanic/Latino and African American/Black in the High Mixed profile. Those who were either laid off or looking for work prior to COVID-19 were over-represented within the Moderate Mixed profile. Additionally, those who were retired, disabled, or otherwise unable to work prior to COVID-19 were over-represented in the predominately positive and predominately negative profile. Finally, high earners ($100,000 or more) were over-represented in the predominately positive profile, whereas, low earners (less than $29,999) were over-represented in the predominately negative profile. Below average ($30,000 to $49,999) and average ($50,000 to $99,999) earners were over-represented in the high mixed profile.

**Table 4:** Demographics of the four latent profiles.

	Latent Profiles			
	Predominantly Positive	Moderately Mixed	Predominantly Negative	High Mixed
	*n* (*% row*)	*n* (*% row*)	*n* (*% row*)	*n* (*% row*)
**Total Sample**	839 (22.49%)	1,748 (46.85%)	849 (22.76%)	295 (7.91%)
**Sex**: χ^2^(3) = 44.797, *p* < .001; Cramer’s V = 0.110, *p* < .001				
Male	422 _a_ (23.06%)	891 _a_ (48.69%)	340 _a_ (18.58%)	177 _a_ (9.67%)
Female	417 _a_ (21.94%)	857 _b_ (45.08%)	509 _b_ (26.78%)	118 _b_ (6.21%)
**Race / Ethnicity**: χ^2^(12) = 111.334, *p* < .001; Cramer’s V = 0.106, *p* < .001				
Caucasian or White	626 _a_ (22.99%)	1243 _a_ (45.65%)	672 _a_ (24.68%)	182 _a_ (6.68%)
Hispanic or Latino	32 _a_ (18.71%)	84 _a,b_ (49.12%)	28 _a,b_ (16.37%)	27 _b,c_ (15.79%)
African American or Black	76 _a_ (23.97%)	137 _a_ (43.22%)	40 _b_ (12.62%)	64 _c_ (20.19%)
Asian American or Asian	71 _a_ (21.07%)	191 _b_ (56.68%)	65 _a,b_ (19.29%)	10 _a_ (2.97%)
Multiracial	34 _a_ (18.58%)	93 _a,b_ (50.82%)	44 _a_ (24.04%)	12 _a,b_ (6.56%)
**Employment prior to COVID-19**: χ^2^(6) = 70.860, *p* < .001; Cramer’s V = 0.097, *p* < .001				
Working	667 _a_ (22.10%)	1418 _a,b_ (46.98%)	650 _a_ (21.54%)	283 _a_ (9.38%)
Laid off or Looking for work	45 _a_ (16.92%)	143 _b_ (53.76%)	70 _a,b_ (26.32%)	8 _b_ (3.01%)
Retired, Disabled or Otherwise not working	127 _b_ (28.41%)	187 _a_ (41.83%)	129 _b_ (28.86%)	4 _b_ (0.89%)
**Annual Income**: χ^2^(9) = 97.76, *p* < .001; Cramer’s V = 0.093, *p* < .001				
Less than $29,999	171 _a_ (18.19%)	457 _a_ (48.62%)	277 _a_ (29.47%)	35 _a_ (3.72%)
$30,000 to $49,999	184 _a,b_ (21.45%)	377 _a_ (43.94%)	197 _b_ (22.96%)	100 _b_ (11.66%)
$50,000 to $99,999	339 _b_ (23.51%)	695 _a_ (48.20%)	272 _b_ (18.86%)	136 _b_ (9.43%)
$100,000 or more	145 _c_ (29.53%)	219 _a_ (44.60%)	103 _b_ (20.98%)	24 _a_ (4.89%)

*Note*. Cramer’s V is used to assess the effect size of the association (0.1 = small; 0.3 = medium; 0.5 = large). Different subscripts indicate a significant difference in the proportion of various demographics (e.g., sex, race, etc.) within each profile with an adjusted Bonferroni alpha (*p* < .05).

Table 5 presents differences in age and psychological distress. The High Mixed profile was associated with being younger and experiencing greater psychological distress.

**Table 5:** Differences in age and psychological distress across latent profiles.

	Predominantly Positive (*n* = 839)		Moderately Mixed (*n* = 1,748)		Predominantly Negative (*n* = 849)		High Mixed (*n* = 295)	
	*M*	*SD*	*M*	*SD*	*M*	*SD*	*M*	*SD*
Age (in years)	42.90 _a_	13.32	38.66 _b_	13.04	39.01 _b_	13.23	35.84 _c_	10.42
Stress (PSS-4; Past Month)	3.91 _a_	3.15	6.33 _b_	3.04	8.81 _c_	3.23	7.78 _d_	1.76
Anxiety (GAD-2; Past Two Weeks)	0.70 _a_	1.22	1.55 _b_	1.55	3.02 _c_	1.88	3.34 _d_	1.55
Depression (PGQ-2; Past Two Weeks)	0.57 _a_	1.06	1.42 _b_	1.51	2.71 _c_	1.76	3.35 _d_	1.44

*Note*. Analyses of variance were conducted for each comparison: **Age**: *F*(3,3727) = 29.94, *p* < .001; *partial η*^*2*^ = .02; **PSS-4**: *F*(3,3727) = 387.46, *p* < .001; *partial η*^*2*^ = .24; **GAD-2**: *F*(3,3727) = 420.08, *p* < .001; *partial η*^*2*^ = .25; **PHQ-2**: *F*(3,3727) = 435.17, *p* < .001; *partial η*^*2*^ = .26. Significant Bonferroni post-hoc difference tests are indicated by different superscripts.

Finally, Table 6 presents the proportion of each profile endorsing specific COVID-19 events. Relative to the other groups, a greater proportion of those in the High Mixed group indicated a loss of wages, having been themselves or knowing someone else that was diagnosed with COVID-19, and knowing someone who died due to COVID-19 complications.

**Table 6:** Proportion of individuals endorsing specific COVID-19 experiences across the four latent profiles.

	Latent Profiles			
	Predominantly Positive	Moderately Mixed	Predominantly Negative	High Mixed
	*n* (*% Profile*)	*n* (*% Profile*)	*n* (*% Profile*)	*n* (*% Profile*)
**Total Sample**	839 (22.49%)	1,748 (46.85%)	849 (22.76%)	295 (7.91%)
*Have you lost wages because of the COVID-19 pandemic*?				
χ^2^(3) = 183.33, *p* < .001; Cramer’s V = 0.222, *p* < .001				
	223 _a_ (26.58%)	649 _b_ (37.13%)	372 _c_ (43.82%)	206 _d_ (69.83%)
*Have you or someone you know been diagnosed with the coronavirus (COVID-19)*?				
χ^2^(3) = 72.383, *p* < .001; Cramer’s V = 0.139, *p* < .001				
	203 _a_ (24.20%)	488 _b_ (27.92%)	249 _b_ (29.33%)	147 _c_ (49.83%)
*Has someone you know died due to complications with the coronavirus (COVID-19)*?				
χ^2^(3) = 172.122, *p* < .001; Cramer’s V = 0.215, *p* < .001				
	72 _a_ (8.58%)	166 _a_ (9.50%)	84 _a_ (9.89%)	102 _b_ (34.58%)

*Note*. All items were responded to dichotomously. Only the percentage of those endorsing the item are reported. Cramer’s V is used to assess the effect size of the association (0.1 = small; 0.3 = medium; 0.5 = large). Different subscripts indicate a significant difference in the proportion of various demographics (e.g., sex, race, etc.) within each profile with an adjusted Bonferroni alpha (*p* < .05).

## Discussion

The present study is the first to investigate the within-individual co-occurrence of negative and positive experiences during the early months of the COVID-19 pandemic among an online, diverse community sample of adults. The first objective was to validate the researcher-designed measure used to assess negative and positive experiences in terms of its factor structure as well as its divergent and convergent validity with markers of psychological distress. The second objective was to identify underlying profiles of negative and positive COVID-19 experiences using a person-centered approach, which provides a more nuanced perspective than the more commonly variable-centered approach prior research has employed. Finally, the third objective sought to better understand these emergent profiles by exploring how they differed in terms of specific stressful events during COVID-19, psychological distress, and demographic information.

Over the past year, the leading narrative, both in research and in the media, on the psychological and mental health consequences of the COVID-19 pandemic has primarily focused on the negative impacts of the pandemic due to increased stress, anxiety, grief, and social isolation measures [5, 14, 15]. However, this narrative was not consistent with the experiences of more than three-quarters of the sample in the present study, who reported at least modest positive experiences during COVID-19. This finding is consistent with a growing body of literature suggesting that COVID-19 experiences may extend beyond negative experiences and include positive experiences as well. Moreover, the present study is the first to apply a person-centered approach to identify four unique profiles of experiences, extending the findings of previous studies that have largely focused on the frequency of negative and/or positive experiences without fully accounting for the individual variability of the COVID-19 experiences or exploring whether or not negative and positive experiences may co-occur within an individual. Furthermore, these studies have either had a non-diverse sample (e.g., primarily female) or have focused on further examining the correlates of negative and positive COVID-19 experiences independently rather than investigating these experiences from a person-centered lens approach. Thus, the present study is unique in exploring concurrent reports of negative and positive experiences in the early onset of the pandemic within a diverse community sample.

This study is also among the first to develop and validate a brief measure assessing the diversity of COVID-19 experiences. As hypothesized, results from an EFA and a CFA revealed a two-factor structure for COVID-19 experiences, with negative and positive experiences emerging as two distinct factors. Further validation of these factors with psychological distress constructs (i.e., stress, anxiety, and depression) suggests that these factors function differently from one another. Specifically, as expected, negative COVID experiences significantly and positively correlated with markers of psychological distress. Similarly, results also indicated that psychological distress constructs were significantly inversely associated with positive experiences, although these relationships were weaker than those with negative experiences. Future research should explore whether positive COVID-19 experiences are in fact associated with concurrent experiences of subjective well-being and vitality [40, 41].

The present findings are additionally consistent with previous literature on both COVID-19 experiences and the divergent relationship between negative and positive emotions indicating that positive experiences, whether COVID-19-specific or not, are weakly yet negatively associated with negative psychological constructs or responses to affective stimuli [42, 43]. This is also in line with positive psychology research on the dual continua of mental health and mental illness in which positive experiences have been found to function in a distinct and different way from negative experiences [44, 45]. Specifically, positive psychology research suggests that the presence of negative experiences (i.e., constructs associated with mental illness such as stress, anxiety, depression) does not inherently suggest an absence of positive experiences (i.e., constructs associated with mental health such as happiness, life satisfaction, wellbeing) and that the complex relationship and interaction between negative and positive experiences merits further investigation [44].

The present study’s profile analysis revealed an interesting and counterintuitive pattern whereby the majority of participants reported a mixed experience of either moderate levels of both negative and positive emotions (46.85%) or high levels of both negative and positive emotions (7.91%). This simultaneous reporting of positive and negative experiences that emerged in the LPA has not been previously documented in COVID-19 literature and challenges the cultural narrative that negative psychological experiences have been predominant during the COVID-19 pandemic [15]. Indeed, only 22.76% of the present sample reported predominantly negative experiences during the early onset of the pandemic. Thus, these findings highlight the importance of using a person-centered approach in investigating experiences during the COVID-19 pandemic and the need to consider both positive and negative experiences in tandem.

Moreover, having established the emergence of four distinct profiles of COVID-19 experiences, the present study sought to better understand these profiles through an investigation of contributing factors including demographics, psychological distress variables, and specific stressful events during COVID-19. To be clear, the small effect sizes, based on Cramer’s V, suggest a cautious interpretation. Nonetheless, some interesting patterns became apparent in the comparison of these profiles. First, participants who reported predominantly negative experiences during the onset of COVID-19 were significantly proportionately more likely to be female, retired, disabled or not working, as well as to report low income. This finding is in line with previous literature [10, 14, 45, 46] which has found that individuals who have reported more negative experiences in response to the onset of COVID-19 have tended to be those already in a position of psychosocial or financial disadvantage. Thus, although they may not have reported experiencing the greatest number of challenges, individuals reporting predominantly negative experiences during COVID-19 in the present study may have been in a greater position of vulnerability at the onset of the pandemic. However, surprisingly, participants who reported predominantly negative experiences during the onset of COVID-19 were not the ones who experienced the highest proportion of stressful events during the early onset of the pandemic.

Interestingly, individuals who reported high levels of both negative and positive COVID-19 experiences were also more likely to have experienced the highest proportion of adverse COVID-19 specific events. Specifically, they were more likely to report loss of wages, having received or knowing someone who received a diagnosis of COVID-19, or having lost someone due to COVID-19 complications. They were also the profile most likely to report symptoms of anxiety and depression and the second most likely to report stress. Thus, although the plethora of challenges caused by the pandemic may have brought about intense negative experiences for these individuals, results suggest that these individuals reported endorsing a high degree of positive experiences during the early months of the COVID-19 as well.

A possible explanation for this discrepancy could be that the high negative and positive experiences reported by individuals in this profile occurred sequentially rather than simultaneously. We hope that future research addresses the temporal order of both negative and positive experiences during COVID-19 as researchers begin to analyze their longitudinal data on the psychological impacts of the pandemic. In the present study, it may have been the case that individuals within the High Mixed profile had good resources to begin with, which may have enabled them to cope well with the high number of challenges that they encountered. This is tentatively supported by the fact that the individuals in this profile were significantly more likely to be working prior to the pandemic and to be reporting an annual income between $30,000 to $99,000.

Alternatively, it may be that the high negative and positive experiences reported during the pandemic occurred simultaneously for these individuals. Although seemingly counter-intuitive, this would be consistent with stress research showing that positive and negative emotions cannot only co-occur during chronic high stress periods but that positive emotions may be a critical part of the stress response in order to foster resilience [47]. Specifically, during periods of high and chronic stress, beyond the natural negative experiences resulting from an adverse situation, individuals may also report positive experiences as a result of trying to form a sense of personal meaning and growth out of the experience [23, 47, 48]. Nevertheless, future longitudinal research is needed in order to elucidate these findings.

In summary, the present study builds upon existing research in the UK in which similar reports of both negative and positive experiences specific to the pandemic were found also using a validated, researcher-designed measure [2]. This communality in finding distinct reports of both negative and positive experiences, despite the fact that the US and UK’s highly politicized national responses to the pandemic were arguably two of the most globally controversial [49], speaks to the underlying strength of this finding. Furthermore, most interestingly, these findings provide a unique contribution by using a person-centered approach to investigate the complexity and diversity of profiles of experiences during the pandemic. These findings document a strong pattern of mixed negative and positive experiences specific to COVID-19, which further strengthens the growing body of literature regarding the complexity of experiences and the surprising potential for resilience during the pandemic.

### Limitations & future directions

The present study is not without limitations. For instance, while its cross-sectional design allowed for a detailed snapshot of individuals’ experiences during the early onset of the pandemic, longitudinal research is needed to elucidate the temporal order of negative and positive experiences during COVID-19, as well as to extend the current findings by considering the potential for adjustment and adaptation in response to stressful events [48, 50], such as the ongoing COVID-19 pandemic. Additionally, while the development and validation of a brief measure of negative and positive experiences during COVID-19 was a novel contribution to the field, further research is needed to potentially expand this measure by considering a broader range of experiences which have been highlighted in the growing body of literature [2, 18]. Similarly, this researcher-developed assessment was validated with self-report measures of psychological distress of which two assessed experiences during the past two weeks (PHQ-2; GAD-2) and one assessed experiences during the past month (PSS-4). As such, future research should seek to further validate this measure with more robust clinical assessments of psychological functioning. Finally, the use of MTURK has its own limitations. First, although the present study’s sample was more economically diverse than previous samples and included a more even sex distribution [2, 18, 23], the sample still suffers from being largely White or Caucasian, a common limitation of MTURK samples. Therefore, the generalizability of the present findings is limited, and research with more ethnically diverse samples is warranted. Second, as noted by one of our reviewers, there is concern within the field as to whether or not the sample characteristics are accurate. Indeed, this is a general limitation with any self-report data including data from university and/or community samples. Nonetheless, data was rigorously screened for completeness, inattention, and suspicious response patterns in order to maintain high data integrity.

## Conclusions

Despite these limitations, the findings in the present study challenge the common narrative, both in research and media reports, that the impacts of COVID-19 have been predominantly negative. In fact, we found that the vast majority of individuals indicated that they had experienced positive experiences in the context of the pandemic. These findings highlight the need for the current discourse on COVID-19 experiences to move beyond a deficit and pathology-oriented model to a salutogenic and strengths-based approach that takes into account human resiliency in the context of the pandemic. In addition, the brief, validated assessment of diverse experiences during COVID-19 that was developed in the present study can henceforth be used, for both research and clinical purposes, to tap both negative and positive experiences simultaneously with a broad range of samples; assessing both gradients of negative and positive impacts of COVID-19 is necessary to ascertain a comprehensive understanding of an individual’s functioning during the pandemic. Furthermore, the emergence of four distinct profiles in the present study also highlights that individuals’ experiences during the early months of COVID-19 have not been unilaterally positive or negative, but rather, highly diverse and undeniably complex. These findings underscore the importance of taking into account the complexity of individuals’ responses to the pandemic in future efforts to quantify the wide range of COVID-19 experiences that may include factors from the area of positive psychology [51]. Lastly, by drawing on data related to psychological distress, COVID-19-specific events, and demographics, the current findings provide novel insights into which individuals have been most at-risk during the pandemic and can inform targeted prevention and intervention for resilience-building during and beyond COVID-19.
